# FcγRIIa (CD32) polymorphism and anti-malarial IgG subclass pattern among Fulani and sympatric ethnic groups living in eastern Sudan

**DOI:** 10.1186/1475-2875-8-43

**Published:** 2009-03-13

**Authors:** Amre Nasr, Nnaemeka C Iriemenam, Hayder A Giha, Halima A Balogun, Robin F Anders, Marita Troye-Blomberg, Gehad ElGhazali, Klavs Berzins

**Affiliations:** 1 Department of Microbiology and Molecular-Biology, Faculty of Science and Technology and Al-Neelain Research Centre, Faculty of Medicine, Al-Neelain University, PO Box 12702, Khartoum, Sudan; 2 Department of Immunology, Wenner-Gren Institute, Stockholm University, SE 10691 Stockholm, Svante Arrhenius väg 16, SE-10 691 Stockholm, Sweden; 3 Department of Medical Microbiology and Parasitology, College of Medicine of the University of Lagos, Idi-araba, PMB 12003 Lagos, Nigeria; 4 Department of Biochemistry, Faculty of Medicine, University of Khartoum, PO Box 102 Khartoum, Sudan; 5 Department of Medical Biochemistry, Faculty of Medicine and Medical Sciences, Arabian Gulf University, PO Box 26671 Manama, Bahrain; 6 Department of Biochemistry, La Trobe University, Victoria 3086, Australia; 7 Department of Clinical Immunology, Pathology and Clinical Laboratory Medicine, Faculty of Medicine, King Fahad Medical City, Riyadh, Saudi Arabia

## Abstract

**Background:**

A SNP at position 131, in the FcγRIIa gene, affects the binding of the different IgG subclasses and may influence the clinical variation seen in patients with falciparum malaria. This study confirms and extends previous findings, analysing the FcγRIIa (CD32) polymorphism in relation to the IgG subclass distribution seen among two sympatric tribes living in eastern Sudan, characterized by marked differences in susceptibility to *Plasmodium falciparum *malaria.

**Methods:**

Two hundred and fifty Fulani subjects living in an area of meso-endemic *P. falciparum *malaria infection were genotyped for the FcγRIIa-131 polymorphism. For comparison, 101 non-Fulani donors – (Masaleit, Hausa and Four) – living in the same study area, were genotyped. The levels of plasma antibodies (IgG and subclasses) to four malaria antigens (AMA-1, MSP 2 – 3D7 & FC27, Pf332-C231) were measured using indirect enzyme-linked immunosorbent assays.

**Results:**

The FcγRIIa-H/H131 genotype was found to be significantly more prevalent in the Fulani as compared to the non-Fulani ethnic groups (36.0% for Fulani versus 17.8% for non-Fulani, adjusted OR 3.10, 95% CI 1.61–5.97, P value < 0.001). The Fulani showed lower anti-malarial IgG1 and IgG3 antibody levels as compared to the non-Fulani and higher levels of IgG2 antibodies.

**Conclusion:**

The FcγRIIa-H/H131 genotype and H131 allele is at higher frequency in the Fulani ethnic group. The H/H131 genotype was consistently associated with higher levels of anti-malarial IgG2 and IgG3 antibodies, while the R/R131 genotype was associated with higher levels of IgG1 antibodies.

## Background

One of the most common causes of morbidity and mortality in African children is *Plasmodium falciparum *malaria [1]. For the last 10 years field studies have been carried out in Daraweesh village in eastern Sudan, aimed at understanding the population dynamics and human immune responses to malaria infections in an area of seasonal and unstable malaria transmission. Studies in Daraweesh have demonstrated that a significant proportion of the population harbours asymptomatic infections detectable by a rise in anti-malarial antibody titres during the transmission season [2-4].

Naturally acquired antibodies are important for protection against asexual blood stages of malaria, as shown by passive transfer of immunoglobulin gamma (IgG) from African malaria-immune adults to Thai malaria-naïve patients [5]. FcγRIIa, one of three receptors for human IgG, is expressed on the surface of all types of cells of the immune system. FcγRIIa is a low-affinity receptor for monomeric IgG, but binds IgG immune complexes efficiently [6]. FcγRIIa is believed to play a major role in eliciting monocyte and macrophage-mediated effector responses against blood-stage malaria parasites. A single nucleotide polymorphism (SNP) G/A, causes an arginine (R) to be replaced with histidine (H) at position 131, defines two allotypes, which differ in their avidity for complexed human IgG2 and IgG3 [7]. The H131 receptor is high-binding for IgG2, while the R131 receptor is low-binding for this subclass. IgG2 is a poor activator of the classical complement pathway, and since FcγRIIa-H131 is essential for handling IgG2 immune complexes therefore this SNP might have an impact on the outcome of the immune response to *P. falciparum *[6].

Previous studies involving the Fulani ethnic group in Burkina Faso and Mali have shown that these individuals are less affected by clinical malaria than individuals from other sympatric ethnic groups [8-13]. In addition, the Fulani in Sudan were significantly less parasitized than the individuals of sympatric non-Fulani ethnic groups [14], corroborating the results previously obtained in Burkina Faso and Mali [8,12,13]. The lower susceptibility to *P. falciparum *malaria seen in the Fulani could, however, not be explained by gene polymorphisms previously associated with malaria resistance, i.e. HbS, HbC, alpha-thal, G6PD and HLA [11,15]. In a longitudinal study of a Fulani population resident in eastern Sudan, an unexpected variation was seen regarding individual disease susceptibility and outbreak severity [16]. In this population, it was recently shown that FcγRIIa (CD32) and Hb AS polymorphisms [17], as well as GM and KM allotypes of IgG, differ significantly between the Fulani and non-Fulani ethnic groups [14]. On the basis of these observations, it may be hypothesized that the FcγRIIa genotype, GM and KM allotypes may contribute to the interethnic differences in malaria susceptibility, possibly in part by influencing the IgG subclass pattern of the anti-malarial antibodies.

In this study, the influence of the FcγRIIa-R/H131 polymorphisms on the IgG subclass patterns of antibodies to four malaria vaccine candidate antigens was analysed in the Fulani and their sympatric non-Fulani ethnic groups in eastern Sudan.

## Methods

### Study area

A detailed description of the study area has been previously reported [16,18,19]. The study was carried out before the rainy seasons between 2004 and 2006 in the Daraweesh village, Gedaref State in eastern Sudan. Daraweesh is 450 km from Khartoum and 16 km from Gedaref town. It is inhabited by approximately 420 Arabic speakers of Fulani ethnic origin whose ancestors settled in this area about a century ago [20]. The village economy is based on agriculture. Malaria transmission is markedly seasonal and unstable and annual peak parasite prevalence ranges from 1 to 40% in different years. The transmission is hypo-endemic and the acquisition of clinical immunity with age is not as obvious as seen in holo-endemic areas and thus malaria remains a problem in all age groups [20]. *Plasmodium falciparum *is responsible for >96% malaria cases, the remainder being *Plasmodium vivax *and *Plasmodium malariae*. *Anopheles arabiensis *is the sole vector.

### Study population

This study is a part of a longitudinal study of infection and immunity to malaria in Daraweesh, which has been going on since 1990. The study received approval from the ethical Committee of the University of Khartoum and national clearance from the Sudanese Ministry of Health. In this Daraweesh population, who are of Fulani origin, a study showed distinctly variable level of disease susceptibility [16]. In the present study, data and samples from a cohort of 250 permanent residents (162 female, 88 male; median of age in 2005, 15.5 years, with the range 4–75 years), monitored clinically and parasitologically by passive case detection for up to 11 years were used. During the malaria transmission season, all individuals feeling unwell reported to the health team that was present in the village on daily basis. A total of 101 subjects from the sympatric non-Fulani ethnic neighbours – Masaleit (51%), Hausa (27.7%) and Four (21.8%) [14], were consecutively selected for the study (60 female, 41 male; median of age in 2005, 17.0 years, with the range 2–55 years). All blood samples were collected before the rainy season and all individuals were asymptomatic and negative for *Plasmodium *parasites.

### Blood collection for genotype and ELISA analysis

Three ml of peripheral blood were collected from the individuals into vacuum EDTA tubes. The collected blood samples were centrifuged for 15 minutes at 250 g. The layer of white cells on top of red blood cells were collected into sterile cryotubes and stored frozen at -20°C for DNA extraction and FcγRIIa genotyping. The plasma was transferred into cryotubes stored at -20°C until use for antibody detection.

### DNA purification

Genomic DNA was purified from the buffy-coat cells using a modified version of the Chelex-100 method and then stored at -20°C [21]. In brief, 25 ml from the cells were incubated overnight at 4°C in 1 ml of 0.5% saponin in 1× phosphate-buffered saline (PBS). The pellet was washed for 30 min with PBS at 4°C, and the supernatant discarded. The pellet was boiled in 120 ml of 5% Chelex-100 in water for 15 min, and the DNA was collected in the supernatants after centrifugation at 250 g for 3 min.

### Genotyping of the FcγRIIa gene

The FcγRIIa genotype was determined using a modified version of a polymerase chain reaction (PCR) method [22]. The PCR conditions were as follows: one cycle at 96°C for five minutes, 30 cycles at 94°C for 30 sec and 56°C for 30 sec, and one cycle at 72°C for 45 sec. The PCR product was digested for 2 h at 37°C using the restriction enzyme *Bst*UI (Fermentas Inc. MD, USA), according to the manufacturer's recommendation. The enzyme cuts at one site in the R131 allele and twice in the H131 allele. The fragments were resolved by electrophoresis in a 2% agarose gel.

### Measurement of antibodies

The levels of serum antibodies (IgG total and subclasses) to four malaria antigens (AMA-1, MSP 2 – 3D7 & FC27, Pf332-C231) were measured using enzyme-linked immunosorbent assays (ELISA), mainly as previously described [23]. Briefly, EIA/RIA plates (Costar, MA, USA) were coated with AMA-1 at 1 μg/ml, 3D7-MSP 2 and FC27-MSP 2 at 1 μg/ml, and Pf332-C231 at 5 μg/ml. The AMA-1 protein, which has an N-terminal hexa-His tag, was expressed in *Escherichia coli *and refolded *in vitro *[24]. Both the 3D7 and FC27 forms of MSP 2 were expressed in *E. coli *with C-terminal hexa-His tags. The expression and purification of these proteins will be described elsewhere (Hobba *et al*, manuscript in preparation). The recombinant Pf332-C231 corresponds to a 231-amino acid fragment in the C-terminal part of Pf332 [25]. The plates were incubated overnight at 4°C, and then blocked for 2 hrs with 0.5% bovine serum albumin (BSA) diluted in carbonate buffer (pH 9.6). Plasma samples diluted in incubation buffer (PBS + 0.5% BSA), 1:1,000 (IgG) and 1:400 (IgG1–4), were added in duplicate and incubated for 1 h at 37°C. The plates were than washed four times, and bound IgG antibodies were detected with goat anti-human IgG-ALP (1:2000) (Mabtech, Nacka, Sweden). IgG subclasses were analysed with their respective biotin conjugated mouse anti-human subclass specific monoclonal antibodies: mouse anti-human IgG1 1:1,000 (M15015, Clone NL16, SkyBio, Bedfordshire, UK), mouse anti-human IgG2 1:3,000 (555874, Pharmingen, Erembodegem, Belgium), mouse anti-human IgG3 1:1,000 (MH 1532, Caltag laboratories, Paisley, UK) and mouse anti-human IgG4 1:2,000 (B3648, Sigma, St. Louis, USA). Alkaline phosphatase (ALP) conjugated streptavidin (Mabtech) diluted 1:2,000 was added to detect bound antibodies of IgG2–4, while ALP-conjugated to goat anti-mouse Ig (Dakopatts, Glostrup, Denmark; 1:1,000) were used for IgG1 antibodies and the plates were developed with nitrophenyl phosphate (Sigma-Aldrich Chemie GmbH, Steinheim, Germany). The absorbance was read at 405 nm using a Vmax™ Kinetic microplate reader (Molecular devices, Menlo Park, USA) and the antibody concentrations were deduced from the log-log correlative coefficient of each IgG subclass standard curve (six dilutions of myeloma proteins of IgG1–4 subclasses), ranging from 0.01 to 3 μg/ml for IgG1, 0.001 to 0.3 μg/ml for IgG2, 0.001 to 0.1 μg/ml for IgG3 and 0.01 to 1 μg/ml for IgG4 according to the manufacturer's recommendation (Biogenesis, Poole, England).

### Statistical analysis

FcγRIIa genotype, allele frequencies and antibody levels were analysed using SPSS version 10.0 (SPSS, Inc, Chicago, IL, USA). Logistic regression analysis was performed with age, sex and antibodies modelled as binary dummy variables (ranked into third distribution using the first third as the reference indicator). Associations were quantified using odds ratios [OR] with 95% confidence intervals [CI] that did not cross 1.00 with P value < 0.05, defined as statistically significant. The FcγRIIa-His/Arg131 group was used as a reference in the analyses, because this genotype is the most prevalent in human populations [26]. An overall comparison of allele frequency using a 2 × 2 chi-square test was performed using the same software.

## Results

A total of 351 individuals were included in the study, 202 (63.2%) females and 129 (36.8) males, with an overall age range of 2 to 75 years. No donor had any malaria parasites detectable by microscopy at the time of sampling; this result was confirmed by PCR screening of the samples (not listed).

### FcγRIIa-R/H131 genotype and allele frequencies in the sympatric ethnic groups

All individuals of the two study groups were genotyped for the FcγRIIa-H/R131 SNP. The H/H131 genotype was at higher frequency in the Fulani than in the non-Fulani ethnic group (36.0% for Fulani versus 17.8% for non-Fulani; unadjusted OR 2.67, 95% CI 1.48–4.83, P < 0.001 and adjusted OR 3.10, 95% CI 1.61–5.97, P < 0.001) (Tables 1 and 2). In contrast, there were no differences between the two groups in the frequency of R/R 131 genotype (17.6% for Fulani versus 20.8% non-Fulani; adjusted OR 0.99, 95% CI 0.52–1.89, P value 0.98) (Tables 1 and 2). However, when using the H/H131 genotype as a reference group in the statistical analysis, the heterozygote H/R131 genotype was found to be statistically significantly at higher frequency in the non-Fulani than in Fulani (46.4% for Fulani versus 61.4% for non-Fulani; adjusted OR 0.34, 95% CI 0.18–0.62, P < 0.001) (Table 2). A similar significant difference was seen for the R/R131 genotype, also being at higher frequency in non-Fulani as compared with Fulani (17.6% for Fulani versus 20.8% for non-Fulani; adjusted OR 0.36, 95% CI 0.17–0.77, P < 0.001) (Table 2).

**Table 1:** Distribution of the FcγRIIa-131 genotypes and allele frequency in the sympatric ethnic groups

	**FcγRIIa genotypes**		
**Ethnic**	**H/H131**	**H/R131**	**R/R131**
Fulani n (%)	90 (36.0%)	116 (46.4%)	44 (17.6%)
Non-Fulani n (%)	18 (17.8%)	62 (61.4%)	21 (20.8%)
			
Allele Frequency*	**H131**	**R131**	

Fulani	0.60	0.40	
Non-Fulani	0.49	0.51	

* 0.60 for Fulani versus 0.40 non-Fulani; OR 1.54, 95% CI (1.09–2.17), P value 0.01

**Table 2:** Logistic regression analysis of FcγRIIa genotypes in Fulani compared with non-Fulani ethnic groups

	***Unadjusted *Odds ratio (95% CI)**	***p *value**	Adjusted* Odds ratio (95% Cl)	***p *value**
H/H131	2.67 (1.48–4.83)	< 0.001	3.10 (1.61–5.97)	< 0.001
R/H131	1.00		1.00	
R/R131	1.12 (0.61–2.05)	0.71	0.99 (0.52–1.89)	0.98
				
H/H131	1.00		1.00	
R/H131	0.37 (0.21–0.68)	< 0.001	0.34 (0.18–0.62)	< 0.001
R/R131	0.42 (0.20–0.87)	0.02	0.36 (0.17–0.77)	0.01

*Adjustment of Odds ratio was performed for sex, age

A genetic difference between the two ethnic groups was also evident when comparing the FcγRIIa-R/H131 allele frequencies, the H131 allele being more common in the Fulani (0.60 for Fulani versus 0.49 non-Fulani; OR 1.54, 95% CI 1.09–2.17, P value 0.01) (Table 1). No such interethnic difference was seen for the R131 allele. The overall genotypic frequencies did not differ statistically among the different age groups within the ethnic groups, neither in the Fulani (P value 0.26) nor in the non-Fulani (P value 0.59). No gender dependent differences in genotype frequencies were seen (P = 0.61).

### IgG antibody reactivities

In this study, antibody responses to four *P. falciparum *blood-stage antigens, AMA-1, MSP2 – 3D7, – FC27 and Pf332-C231, were analysed in the Fulani and the non-Fulani ethnic groups. There were no statistically significant differences in anti-malarial total IgG antibody levels between the ethnic groups, and therefore the following analyses were limited only to the IgG subclasses. In general, IgG1 and IgG3 subclass antibodies were found to be at higher levels than IgG2 and IgG4 antibodies in both ethnic groups. However, the antibody levels varied for the different antigens. When comparing the antibody levels between the two ethnic groups, the non-Fulani showed significantly higher levels of IgG1 antibodies reactive with AMA-1 (P < 0.001), and also tended to have higher IgG3 antibody levels (P = 0.062) (Table 3). The IgG2 antibody responses reactive with AMA-1 were, however, statistically significantly higher in the Fulani than in the non-Fulani ethnic group (P = 0.024) (Table 3). While antibodies reactive with MSP2-3D7 showed a similar pattern of interethnic differences in IgG subclass levels as those reactive with AMA-1, IgG1 antibodies reactive with MSP2-FC27 were at higher levels in the Fulani as compared to the non-Fulani (P = 0.041) (Table 3). Regarding Pf332-C231 reactive antibodies, IgG3 and IgG4 subclasses were significantly higher in the non-Fulani than in the Fulani (P < 0.001) (Table 3). No differences in levels of antibodies of the different IgG subclasses were seen among the different ethnicities within the non-Fulani group.

**Table 3:** Logistic regression analysis of immunoglobulin classes in Fulani and Non-Fulani ethnic groups

**Antigen**	**IgG subclass**	**OR^† ^(95% CI)**	***p *value**
AMA-1	IgG1	1.2 (1.1 to 1.3)	< 0.001
	IgG2	0.6 (0.3 to 0.9)	0.024
	IgG3	2.2 (0.9 to 5.4)	0.062
	IgG4	0.4 (0.1 to 1.2)	0.113
			
3D7-MSP2	IgG1	1.1 (1.0 to 1.2)	0.015
	IgG2	0.6 (0.4 to 0.9)	0.018
	IgG3	1.0 (1.0 to 1.0)	0.069
	IgG4	0.1 (0.05 to 0.3)	< 0.001
			
FC27-MSP2	IgG1	0.5 (0.2 to 0.9)	0.041
	IgG2	2.9 (1.2 to 7.1)	0.016
	IgG3	2.2 (1.4 to 3.3)	< 0.001
	IgG4	0.5 (0.2 to 1.3)	0.159
			
Pf332-C231	IgG1	2.5 (0.8 to 6.8)	0.081
	IgG2	1.0 (0.9 to 1.0)	0.978
	IgG3	2.7 (1.6 to 4.3)	< 0.001
	IgG4	8.1 (3.5 to 18.6)	< 0.001

^† ^OR represent odds ratios while CI represents confidence intervals. Fulani were assigned 0 while non-Fulani were assigned 1 in the logistic regression analysis. OR above 1 represented value associated to non-Fulani while less than 1 value represented Fulani.

### FcγRIIa genotype and *P. falciparum*-specific IgG subclass distribution

In order to see if the FcγRIIa genotype affected the specific IgG subclass levels, using four different malarial antigens, the relationship between FcγRIIa genotype and IgG antibody subclasses was analysed. For the study population as a whole, including both the Fulani and the non-Fulani individuals, the H/H131 genotype was statistically significantly associated with higher levels of IgG2 (P < 0.001), IgG3 (P = 0.01) and IgG4 antibodies against AMA-1 (P = 0.01) (Table 4). Furthermore, there was a trend for an association of the R/R131 genotype with higher levels of IgG1 antibodies against AMA-1 (P = 0.05) (Table 4). Interethnic comparison of AMA-1 reactive antibodies in relation to FcγRIIa genotypes showed that non-Fulani H/H 131 carriers had significantly higher levels of IgG1 (adjusted OR 0.04, 95% CI 0.02–0.12; P < 0.001) and IgG3 (adjusted OR 0.31, 95% CI 0.17–0.57; P < 0.001) (Table 5). No such interethnic differences were found for the IgG2 and IgG4 antibody subclasses.

**Table 4:** Logistic regression analysis of FcγRIIa-H/R131 genotypes in relation to anti-malarial IgG subclasses in the combined study populations

**Anti-malarial antibodies**	**FcγRIIa-H/R131 genotypes**	**OR^† ^(95% CI)**	***p *value**
**AMA-1**			
IgG1	H/H	1.15 (0.57–2.31)	0.70
	H/R	1	
	R/R	1.91 (1.00–3.64)	**0.05**

IgG2	H/H	2.65 (1.43–4.91)	**< 0.001**
	H/R	1	
	R/R	1.34 (0.69–2.61)	0.38

IgG3	H/H	2.26 (1.21–4.22)	**0.01**
	H/R	1	
	R/R	1.38 (0.70–2.72)	0.35

IgG4	H/H	2.93 (1.33–6.41)	**0.01**
	H/R	1	
	R/R	1.15 (0.53–2.48)	0.72

**3D7-MSP2**			
IgG1	H/H	0.10 (0.03–0.20)	**< 0.001**
	H/R	1	
	R/R	0.86 (0.41–1.80)	0.69

IgG2	H/H	2.76 (1.32–4.47)	**< 0.001**
	H/R	1	
	R/R	1.27 (0.66–2.46)	0.48

IgG3	H/H	2.37 (1.28–4.38)	**0.01**
	H/R	1	
	R/R	1.21 (0.62–2.35)	0.58

IgG4	H/H	2.40 (1.29–4.45)	**0.01**
	H/R	1	
	R/R	1.47 (0.74–2.92)	0.27

**FC27-MSP2**			
IgG1	H/H	1.26 (0.65–2.43)	0.50
	H/R	1	
	R/R	2.38 (1.29–4.39)	**0.01**

IgG2	H/H	2.37 (1.28–4.38)	**0.01**
	H/R	1	
	R/R	1.21 (0.62–2.35)	0.58

IgG3	H/H	2.86 (1.50–5.46)	**< 0.001**
	H/R	1	
	R/R	1.45 (0.70–3.02)	0.32

IgG4	H/H	2.46 (1.33–4.55)	**< 0.001**
	H/R	1	
	R/R	1.77 (0.66–2.48)	0.47

**Pf332-C231**			
IgG1	H/H	1.33 (0.68–2.59)	0.40
	H/R	1	
	R/R	2.43 (1.31–4.50)	**0.01**

IgG2	H/H	2.26 (1.21–4.22)	**0.01**
	H/R	1	
	R/R	1.38 (0.70–2.72)	0.35

IgG3	H/H	2.44 (1.32–4.51)	**< 0.001**
	H/R	1	
	R/R	1.18 (0.61–2.30)	0.62

IgG4	H/H	1.79 (0.92–3.49)	0.10
	H/R	1	
	R/R	1.33 (0.59–2.97)	0.50

**Table 5:** Logistic regression analysis of FcγRIIa-H/R131 genotypes in relation to anti-malarial IgG subclasses in Fulani compared with non-Fulani groups

**Anti-malarial antibodies**	**FcγRIIa-H/R131 genotypes**	**OR^† ^(95% CI)**	***p *value**
**AMA-1**			
IgG1	H/H	0.04 (0.02–0.12)	**< 0.001**
	H/R	1	
	R/R	0.86 (0.42–1.78)	0.69

IgG2	H/H	0.70 (0.35–1.37)	0.30
	H/R	1	
	R/R	0.61 (0.34–1.11)	0.10

IgG3	H/H	0.31 (0.17–0.57)	**< 0.001**
	H/R	1	
	R/R	0.69 (0.34–1.38)	0.30

IgG4	H/H	0.59 (0.30–1.15)	0.12
	H/R	1	
	R/R	0.90 (0.41–1.95)	0.78

**3D7-MSP2**			
IgG1	H/H	0.10 (0.03–0.20)	**< 0.001**
	H/R	1	
	R/R	0.86 (0.41–1.80)	0.69

IgG2	H/H	2.43 (1.32–4.47)	**< 0.001**
	H/R	1	
	R/R	0.83 (0.41–1.69)	0.61

IgG3	H/H	0.49 (0.26–0.91)	**0.03**
	H/R	1	
	R/R	0.81 (0.40–1.63)	0.55

IgG4	H/H	2.76 (1.45–5.26)	**< 0.001**
	H/R	1	
	R/R	0.59 (0.28–1.25)	0.17

**FC27-MSP2**			
IgG1	H/H	0.53 (0.28–1.01)	0.10
	H/R	1	
	R/R	0.86 (0.42–1.78)	0.68

IgG2	H/H	0.45 (0.24–0.85)	**0.01**
	H/R	1	
	R/R	0.70 (0.35–1.37)	0.30

IgG3	H/H	0.13 (0.10–0.26)	**0.01**
	H/R	1	
	R/R	0.76 (0.37–1.57)	0.46

IgG4	H/H	0.70 (0.36–1.39)	0.31
	H/R	1	
	R/R	0.97 (0.54–1.74)	0.92

**Pf332-C231**			
IgG1	H/H	1.25 (0.67–2.30)	0.48
	H/R	1	
	R/R	0.72 (0.36–1.44)	0.36

IgG2	H/H	0.22 (0.11–0.44)	**< 0.001**
	H/R	1	
	R/R	0.69 (0.34–1.38)	0.29

IgG3	H/H	4.25 (2.28–7.91)	**< 0.001**
	H/R	1	
	R/R	0.91 (0.45–1.84)	0.80

IgG4	H/H	0.10 (0.03–0.20)	**< 0.001**
	H/R	1	
	R/R	0.69 (0.26–1.42)	0.25

Regarding antibodies reactive with MSP2-3D7 or -FC27 in the study population as a whole, a similar pattern as with AMA-1 reactive antibodies was seen. The H/H 131 genotype carriers showed statistically significantly higher levels of IgG2 (P < 0.001 and = 0.01, respectively), IgG3 (P = 0.01 for both antigens) and IgG4 (P = 0.01 and < 0.001, respectively), while the R/R 131 genotype carriers showed higher levels of IgG1 antibodies (P = 0.01 for both antigens) (Table 4).

Interethnic comparison of MSP2-3D7 reactive antibodies in relation to FcγRIIa genotypes showed that non-Fulani H/H 131 carriers had significantly higher levels of IgG1 (adjusted OR 0.10, 95% CI 0.03–0.20; P < 0.001) and IgG3 (adjusted OR 0.49, 95% CI 0.26–0.91; P value 0.03), while Fulani H/H 131 carriers showed higher levels of IgG2 (adjusted OR 2.43, 95% CI 1.32–4.47; P < 0.001) and IgG4 antibodies (adjusted OR 2.76, 95% CI 1.45–5.26; P < 0.001) (Table 5). In contrast, an interethnic comparison for MSP2-FC27 reactive antibodies, showed that the non-Fulani H/H 131 genotype carriers had significantly higher levels of IgG2 (adjusted OR 0.45, 95% CI 0.24–0.85; P = 0.01) and IgG3 (adjusted OR 0.13, 95% CI 0.10–0.26; P < 0.001) than the Fulani, while no significant interethnic differences were found regarding IgG1 and IgG4 antibody levels with any of the genotypes (Table 5).

Regarding antibodies reactive with Pf332-C231 in the study population as a whole, the H/H131 genotype was statistically significantly associated with higher levels of IgG2 (P = 0.01) and IgG3 (P < 0.001) (Table 4). Furthermore, the R/R131 genotype was statistically significantly associated with higher levels of IgG1 antibodies reactive with this antigen (P = 0.01) (Table 4).

Interethnic comparison of Pf332-C231 reactive antibodies in relation to FcγRIIa genotypes showed that non-Fulani H/H 131 carriers had significantly higher levels of IgG2 (adjusted OR 0.22, 95% CI 0.11–0.44; P < 0.001) and IgG4 (adjusted OR 0.10, 95% CI 0.03–0.20; P < 0.001), IgG3 antibodies were at significantly higher levels in H/H 131 carriers of the Fulani group (adjusted OR 4.25, 95% CI 2.28–7.91; P < 0.001) (Table 5).

## Discussion

The present study confirms and extends the previous finding in the same study population, that the prevalence of the FcγRIIa-H/H131 genotype and the H131 allele are statistically significantly higher in Fulani than in sympatric non-Fulani children [17]. The prevalence of both the FcγRIIa-H/H131 genotype and the H131 allele was similar in the different age groups of the Fulani, indicating that the H131 allele carriage has no evident influence on the survival of the individuals. Interestingly, a similar higher frequency of the H/H131 genotype and H131 allele was recently observed also in the Fulani living in Mali, compared to the sympatric ethnic group, the Dogon [27]. It is well established that the Fulani in Mali and Burkina Faso are less affected by malaria than other sympatric ethnic groups, the Dogon and the Mossi/Rimaibé, respectively [12]. Similarly, the Fulani in the present study site has been shown to have lower *P. falciparum *parasite densities and parasite rates than the neighbouring ethnic group, the Masaleit [17]. Whether the FcγRIIa-R/H131 polymorphism is a contributing factor in the observed ethnic differences in susceptibility to malaria remains to be studied in different epidemiological settings.

Contrasting results have been reported regarding associations of FcγRIIa-R/H131 genotypes with malaria disease outcomes [6]. Thus, one study observed an association between the H/H131 genotype and susceptibility to severe malaria [28], while others found R/R131 genotype carriers to be less likely to be at risk for high density *P. falciparum *infection, compared to the R/H131 heterozygotes [1,29]. Another previous report has associated the H/H131 genotype with protection against malaria [1]. However, contrasting results also with regard to the R/R131 genotype and protection from malaria have been reported [28-31]. The FcγRIIa-R/R131 genotype in Sudanese patients was recently shown to be associated with higher risk to develop severe malaria [32], while in an Indian population, the H/H131 genotype was significantly associated with protection from malaria disease manifestation [33]. The discrepant results in this context may most likely be explained by genetic differences between the populations studied, as well as differences in gene-environment interactions and in patterns of malaria transmission in the different study areas.

Several studies in Burkina Faso and Mali have shown that the Fulani have significantly higher levels of anti-malarial antibodies than other sympatric ethnic groups [9,10,13], including antibodies of all the IgG subclasses, except IgG4 [8]. In contrast, in the present study, the non-Fulani had significantly higher levels of anti-malarial IgG1 and IgG3 antibodies than the Fulani for three of the four antigens studied, but not for MSP2-FC27. The Fulani, tended to have significantly higher levels of anti-malarial IgG2 antibodies than the non-Fulani group. These discrepant results seen in Burkina Faso, Mali and Sudan may most likely be explained by differences in patterns of malaria transmission in the different study areas [14,16-19]. It should be recalled that Sudan is a country characterized by hypo/meso-endemicity, with seasonal and unstable malaria transmission, unlike Burkina Faso and Mali, characterized with hyper/holo-endemic malaria transmission [8]. In Daraweesh an entomological inoculation rate (EIR) of one infective bite/year has been estimated based on surveys in drought-free years [34]. Almost all malaria cases in Daraweesh are clinically uncomplicated, probably because of continuous monitoring by the health team that visits the village on a daily basis and provides free drug treatment [16,19].

While the FcγRIIa-R131 molecule shows a similar binding of the cytophilic subclasses IgG1 and IgG3 [35,36], the H131 receptor binds IgG3 more efficiently and is the only FcγR that binds IgG2 efficiently [7,37-40]. Thus, in the presence of the FcγRIIa-H131 receptor, IgG2 antibodies should also be considered as cytophilic, and thus this receptor is essential for effective IgG2-mediated cellular activation. The present study indicates that the differential recognition of the IgG subclasses by the R131 and H131 FcγRIIa may have an impact on the pattern of IgG subclasses in the anti-malarial immune response. Higher levels of IgG2 and IgG3 antibodies were associated with the H/H genotype, while higher levels of IgG1 antibodies were associated with the R/R131 genotype. In line with this, the Fulani showed a higher frequency of the FcγRIIa-H131 allele and higher levels of anti-malarial IgG2 antibodies than sympatric non-Fulani ethnic groups. A similar higher frequency of the FcγRIIa-H131 allele and a higher proportion of IgG2 among the anti-malarial antibodies, were recently observed in the Fulani in Mali compared to a sympatric non-Fulani ethnic group [27].

There is accumulating evidence for IgG1 and IgG3 antibodies playing an important role in protection against malarial disease [41]. However, the contribution of parasite-reactive IgG2 antibodies in protection against clinical malaria [32,42] and/or in susceptibility to disease [35,43] has been indicated in some epidemiological settings. In Burkina Faso, the association between anti-malarial IgG2 antibodies and protection against malaria, was suggested to be due to the relatively high prevalence of the IgG2 binding H/H131 genotype in that study population [42]. Whether the higher levels of anti-malarial IgG2 antibodies and the higher frequency of the FcγRIIa-H131 allele in the Fulani contributes to the lower susceptibility to malaria in this ethnic group as compared to sympatric non-Fulani ethnic groups, remains to be investigated in extended epidemiological studies.

## Conclusion

The present study confirms and extends the previous findings that the FcγRIIa-H/H131 genotype and the H131 allele are more frequent in the Fulani, than in their sympatric ethnic neighbours. The Fulani in Sudan had lower anti-malarial IgG1 and IgG3 antibody levels than the non-Fulani and higher levels of IgG2 antibodies. The higher IgG2 antibody levels seen in the Fulani may be due to the relatively high prevalence for the H/H131 genotype in this group. Further studies are needed to elucidate if the FcγRIIa-R/H131 polymorphism may be a contributing factor to the differential susceptibility to malaria seen in different ethnic groups.

## Competing interests

The authors declare that they have no competing interest.

## Authors' contributions

AN carried out the sampling, the genotyping of FcγRIIa polymorphism and participated in the statistical analysis. NCI performed the ELISA and also participated in the statistical analysis. Both AN and NCI drafted the manuscript. GE, KB, HG and MT designed the study and set up the framework, financed and revised the manuscript. HAB and RFA prepared and provided the antigens used in the study and participated in the manuscript preparation. All authors read and approved the final version of the manuscript.
